# Foliar Spray of Fe-Asp Confers Better Drought Tolerance in Sunflower as Compared with FeSO_4_: Yield Traits, Osmotic Adjustment, and Antioxidative Defense Mechanisms

**DOI:** 10.3390/biom10091217

**Published:** 2020-08-21

**Authors:** Qasim Ali, Shafaqat Ali, Mohamed A. El-Esawi, Muhammad Rizwan, Muhammad Azeem, Abdullah Ijaz Hussain, Rashida Perveen, Mohamed A. El-Sheikh, Mohammed Nasser Alyemeni, Leonard Wijaya

**Affiliations:** 1 Department of Botany, Government College University Faisalabad, Faisalabad 38000, Pakistan; mazeem@gcuf.edu.pk; 2 Department of Environmental Sciences and Engineering, Government College University, Allama Iqbal Road, Faisalabad 38000, Pakistan; mrazi1532@yahoo.com; 3 Department of Biological Sciences and Technology, China Medical University, 40402 Taichung, Taiwan; 4 Botany Department, Faculty of Science, Tanta University, Tanta 31527, Egypt; mohamed.elesawi@science.tanta.edu.eg; 5 Department of Chemistry, Government College University Faisalabad, Faisalabad 38000, Pakistan; abdullahijaz@gcuf.edu.pk; 6 Department of Physics, University of Agriculture Faisalabad, Faisalabad 38000, Pakistan; 2007ag942@uaf.edu.pk; 7 Department of Botany and Microbiology, College of Science, King Saud University, Riyadh 11451, Saudi Arabia; melsheikh@ksu.edu.sa (M.A.E.-S.); mnyemeni@ksu.edu.sa (M.N.A.); leon077@gmail.com (L.W.)

**Keywords:** water relations, seed yield, antioxidative defense mechanism, amino acids, nutrient uptake

## Abstract

Different techniques are being employed to reduce the adverse effects of water stress on seed yield and quality of crop plants. The current study aimed to improve the water stress tolerance of field-grown sunflower by foliar-supplied ecofriendly iron-chelated aspartate (Fe-Asp) in comparison with FeSO_4_. Water stress decreased the plant growth and yield, accompanied with disturbed water relations, nutrient acquisition, accumulation of amino acids, and antioxidative defense mechanisms. However, lipid peroxidation, total anthocyanin, and photosynthetic pigments were increased. Fertigation of FeSO_4_ and Fe-Asp as foliar sprays proved effective to reduce the negativities of limited irrigation on biomass production and seed yield, accompanied with a reduction in lipid peroxidation and improvements in water relations, antioxidative defense mechanisms, and leaf photosynthetic pigments. In comparison with FeSO_4_, foliary applied Fe-Asp better improved the plant water relations with more accumulation of essential amino acids and nutrient acquisition, especially leaf aspartate (Asp) and Fe accumulation which showed better translocation. Overall, foliary applied Fe-Asp proved better for induction of drought tolerance in sunflower plants as compared with FeSO_4_. The study recommended the use of the ecofriendly Fe-Asp as a foliar spray for better growth and production of sunflower under limited irrigation.

## 1. Introduction

Among different abiotic factors, shortage of high-quality fresh water for irrigation is the most threatening one in the present scenario [1,2]. It is also a major hindrance that researchers are facing in fulfilling world food demand along with the malnutrition issue. Under water deficit conditions, plants grow under disturbed cellular water relations with altered physiological processes, reduced nutrient uptake, increased lipid peroxidation, disturbed photosynthetic efficiency, and reduced growth and seed yield [1,3,4,5].

To tolerate the various environmental stress conditions, plants evolve some adaptations to maintain the cellular water relations for better photosynthetic efficiency, assimilation, and final production [6,7]. However, most of the high yielding crop varieties are not drought-tolerant and do not have efficient mechanisms to tolerate such environmental conditions. To better produce such high yielding genotypes, different techniques have been employed, including the use of better agronomic practices, molecular genetic markers, and exogenous application of organic and inorganic chemicals [8,9,10]. The foremost impact of water shortage is the reduction of nutrient uptake from soil, as the low amount of water in soil reduces nutrient uptake due to the reduced replenishment of nutrients in soil medium as a result disturbed ion homeostasis at cellular levels [1,11,12]. Its severity becomes more problematic when agricultural lands lack essential nutrients. Among different micro and macro essential nutrients, Fe deficiency in most of the soils is also a serious issue that becomes very severe under water deficit conditions [13,14,15].

Iron has a prime importance among different minerals due to its vital functions in different plant physio-biochemical processes [14,16]. Fe is involved in the biosynthesis of chlorophyll molecules and the regulation of different enzyme functions [17]. In plants, Fe represents an integral part of heme proteins, such as cytochromes, as well as non-heme proteins, especially ferredoxin [18]. Cytochromes are part of electron transfer systems in chloroplasts and mitochondria. Moreover, Fe also plays an important role in regulating the activities of catalases and peroxidases which have essential roles in antioxidant defense mechanisms [19].

Soil contains Fe as ~1–5% of the total silicate minerals. Iron oxides and hydroxides are the forms of Fe mostly available in the soil. High soil pH, ranging from 7.4 to 8.5, is a major cause of deficiency, especially under water deficit conditions [20]. Iron deficiency symptoms initially include yellowing of younger leaves between the veins as well as browning of leaves at margins under severe deficiency, resulting in white necrotic areas and premature leaf drops [21,22,23]. Other major causes of Fe deficiencies in plants are soil erosion [24] and high soil moisture, which result in dieback of twigs and branches [25]. Fe-deficient plants are also susceptible to damage by the attack of even weak pathogens [26]. Under severe Fe deficiencies, reduction in the de novo synthesis of chlorophyll ultimately decreases the rate of photosynthesis, resulting in a severe reduction in growth and seed yield [27].

To combat Fe deficiency, fertilizers such as FeCl_2_ and FeSO_4_ are being used through soil application or foliar spray [28,29]. Although FeSO_4_ is widely used as a fertilizer, its use has some drawbacks due to its inorganic nature and the negative effects on plants it may cause [30]. Furthermore, the content of Fe in plants decreases due to the oxidation reactions taking place in the cell when it is applied in inorganic form [31]. Nowadays, application of ecofriendly biochemicals, such as chelates, is required to replace the inorganic fertilizers in order to meet the plant essential nutrient requirements. Like other chelates, amino acid-chelated micronutrients are gaining more interest for meeting the plant micronutrient requirement as well as for improving plant stress tolerance for better yield and nutritional quality. It is found that in chelation uptake, mobility and availability of micronutrient increase without oxidation reactions. Additionally, in the case of micronutrient amino acid chelation, both parts of chelation proved beneficial for cellular metabolic activities [32]. The amino acid metabolism also plays important roles in improving plant tolerance to abiotic stresses via regulating different cellular metabolic activities [7,32]. Among different amino acids that play significant roles in surviving of plants under abiotic stresses, Asp is also of prime importance [33]. It belongs to a group of amino acids, namely, the Asp family, and is a precursor of other essential amino acids including isoleucine, methionine, and threonine. For maintaining the cellular pH, it acts as buffering agent [34]. The biosynthetic processes including protein and chlorophyll are regulated by aspartic acid. Moreover, it acts as a precursor of several antioxidants, enzyme cofactors, and vitamins. It is also involved in cellular reduction homeostasis and defense mechanisms [35].

To date, and to the best of our knowledge, very few or no reports are available about the use of ecofriendly chelated Fe-Asp to overcome plant Fe deficiency and to improve water stress tolerance for better seed yield and nutritional quality. Though the use of FeSO_4_ as an exogenous application is considered beneficial to overcome plant Fe deficiency, its negative impacts are also reported on plants and environment due to its chemical nature. Therefore, the hypothesis of the present study was that Fe-chelated Asp might comparatively be more beneficial to overcome the plant Fe deficiency and to improve the water stress tolerance of sunflower (*Helianthus annuus* L.) plants when it is applied foliary.

Among oilseeds, sunflower is the most important crop and it is well adapted to the agroecological conditions of different regions worldwide. Its oil is used to prevent heart diseases, as it is rich in polyunsaturated fatty acids and vitamins [36,37]. However, its production is threatened due to the shortage of fresh water for irrigation under changing environmental conditions [38,39]. Many developing countries are under threat due to changing global environmental conditions, whereas the majority of their cultivatable area is rainfed [40], and the danger of drought is being considered serious. Therefore, new techniques are required for better production to combat water shortage.

The objective of the present study was to investigate the key role of Fe-chelated Asp in improving the tolerance of sunflower to water stress via investigating different growth and yield attributes, water relations, antioxidative defense mechanism, uptake of nutrient including Fe, and the alterations in the levels of amino acids belonging to Asp family. Additionally, the uptake and translocation of Fe and Asp were also investigated.

## 2. Material and Methods

### 2.1. Study Area

Experiments were conducted in the field research area of the Department of Botany located in the New Botanical Garden of Government Collage University Faisalabad Pakistan (latitude 30°30′ N, longitude 73°10′ E and altitude 213 m), under open natural conditions. In the crop sowing season, the land was irrigated well.

### 2.2. Experimental Design

The design of the experiment was split plot in factorial arrangement. Two main plots of the research area were made, specified to each specific level of water stress. Based on the number of foliar treatments, each main plot was further subdivided in to six sub-plots. Different foliar treatments used 0.25% and 0.5% levels of Fe-Asp and FeSO_4_, including no spray (NS) and water spray (WS). Each subplot comprised three rows of equal size with a row-to-row distance of 24 inches. Each row was corresponding to each specific replicate of each treatment. The seeds of sunflower cultivar Sun Cross were hand sown. Seeds were purchased from Ayoub Agricultural Research Institute (AARI) Faisalabad, Pakistan. Before sowing, the seed germination potential test was performed under growth room conditions in Petri dishes. The number of irrigations was used as the water stress treatment. After 10 days of germination, the first irrigation was applied to all the plots. Then, thinning was performed to maintain a plant-to-plant distance of 30 cm. The main plot allocated for water stress treatment was irrigated only twice during whole growth period i.e., at vegetative and reproductive stages only. However, the other main plot was allocated as non-stressed, and irrigated as per the irrigation requirements. Foliar spray of different treatments was done after 21 days of the onset of water stress treatment. Foliar spray was done in evening before sunset. For the maximum absorption, in each prepared solution, 0.1% tween-20 was added as a surfactant. After three weeks of the foliar spray, data were collected for various attributes. Fresh leaf material for the estimation of various biochemical analyses was collected using liquid nitrogen and stored at −80 °C for the laboratory analyses. Uniform plants from each treatment were up rooted to measure the different growth attributes. After measuring fresh biomass of roots and shoots, the same plants were oven-dried to estimate their dry biomass.

### 2.3. Physico-Chemical Properties of Experimental Soil

For the estimation of soil physico-chemical properties, soil from the experimental site was oven-dried, ground well, and used for different analysis. The methods as described by Davis and Freitas [41] were followed for the estimation of pH and EC, while the soil saturation percentages of ${\mathrm{SO}}_{4}^{-2}$, ${\mathrm{CO}}_{3}^{-2}$, and ${\mathrm{HCO}}_{3}^{-2}$ in soil solution were determined following the method of Estefan et al. [42]. Available P and N were estimated following Olsen and Sommers [43] and Nelson and Bremner [44], respectively. The method described by Page et al. [45] was used for the determination of organic matter, while Na and SAR were estimated following Knudsen et al. [46]. The estimation of Cl^−^ in soil solution was made using the chloride analyzer. However, the estimation of Ca and Mg in the soil extracts was done following Nelson [47]. The average EC and pH of the soil was 2.53 ds·m^−1^ and 7.8, respectively. The soil had a saturation percentage (34%), available total P (8.6 ppm), and N (0.73%). The soil was sandy loam having organic matter (1.15%). The soil solution has SAR (0.086 meq L^−1^), Na (2.98 meq L^−1^), Ca^2+^ + Mg^2+^ (14.3 meq L^−1^), Cl^−^ (8.52 meq L^−1^), SO_4_^−2^ (1.98 meq L^−1^), soluble ${\mathrm{CO}}_{3}^{2-}$ (traces), and HCO_3_^−^ (4.93 meqL^−1^).

### 2.4. Preparation of Chelation

**Figure 1:**
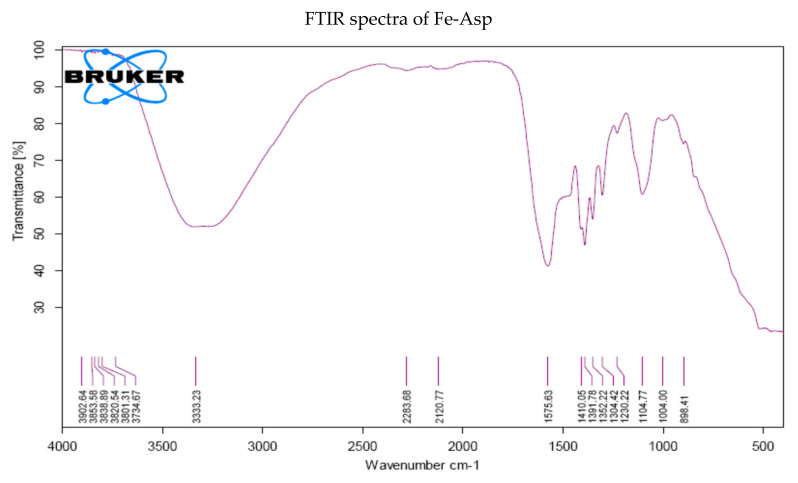

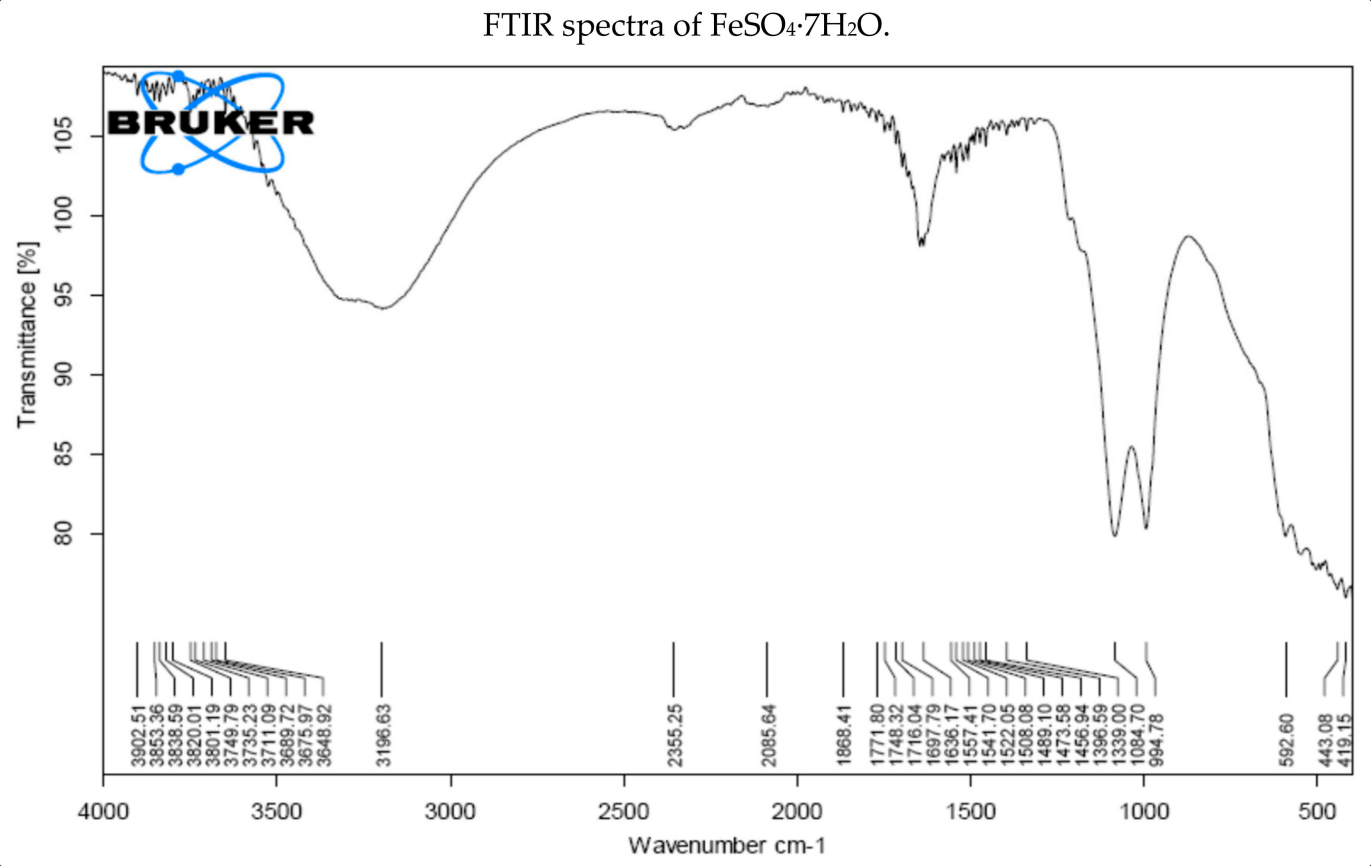
FTIR spectra of Fe-chelated aspartate and FeSO_4_·7H_2_O.

For the preparation of Fe-chelated aspartate (Fe-Asp), the method ascribed by Leu [48] was followed. Briefly, 260 g FeSO_4_·7H_2_O was added and dissolved in dH_2_O. Then, L-aspartate monohydrochloride (146.12 g) was added and warmed well at 95 °C for 3 h. The chelation was confirmed by FTIR analysis as shown in Figure 1.

### 2.5. Leaf Area Estimation

For the estimation of leaf area, the method ascribed by Kvet and Marshall [49] was followed. Fully mature leaf from the top was used to estimate its maximum width and length. After multiplying the length and width, the result was multiplied by 0.75, a common constant factor for the dicots. 

### 2.6. Determination of Leaf Relative Water Content (LRWC)

For the estimation of LRWC, the method ascribed by Sade et al. [50] was followed. Fully developed third leaf from top was excised using a scissor, marked with a permanent marker, and the fresh weight (FW) was measured using an electric balance. The leaves were then dipped for 6 h in distilled water. Then, the turgid weights (TW) of the leaves were recorded after absorbing the excess water on leaf surface using a blotting paper. These leaves were then dried in an electric oven at 70 °C for 48 h and the dry weight (DW) was recorded. The LRWC was also measured using the equation given below.

(1) $$\mathrm{LRWC}\;\left(\%\right)=\;\frac{\mathrm{Leaf}\;\mathrm{FW}-\mathrm{Leaf}\;\mathrm{DW}}{\mathrm{Leaf}\;\mathrm{TW}-\mathrm{Leaf}\;\mathrm{DW}}\times 100$$

### 2.7. Determination of Relative Membrane Permeability

For the estimation of leaf relative membrane permeability (LRMP), the fully matured third leaf from top was used following the method of Yang et al. [51]. The excised leaf was cut into small pieces (1 cm). 0.5 g of leaf was homogenized in 20 mL deionized dH_2_O. Then, the EC of the assayed material was measured after vortexing for 5 s, and was termed it as EC0. The EC of the assayed material was measured again after storing the material at 4 °C for 24 h and was termed as EC1. The assayed material was then autoclaved at 120 °C for 30 min. Then, the EC of the material was measured again after cooling at room temperature and was termed it as EC2. The following equation was then used to measure the LRMP.

(2) $$\mathrm{Leaf}\;\mathrm{relative}\;\mathrm{membrane}\;\mathrm{permeability}\;\left(\%\right)=\frac{\mathrm{EC}1-\mathrm{EC}0}{\mathrm{EC}2-\mathrm{EC}0}\times 100$$

### 2.8. Quantification of Leaf Photosynthetic Pigments

Leaf photosynthetic pigments such as chlorophyll (Chl.) *a*, Chl. *b*, Chl *a*/*b*, and total Chl. were quantified following the method of Arnon [52], and leaf carotenoid (Car) content was measured following the method of Kirk and Allen [53]. Acetone (80%) was used for the extraction of photosynthetic pigments. Briefly, 0.1 g chopped leaf material was mixed in 10 mL acetone and then kept at 4 °C for 24 h. The material was then filtered and the absorbance was read using the spectrophotometer (Hitachi U-2001, Tokyo, Japan) at three wavelengths, i.e., 663, 645, and 480 nm. The equations given below were used to measure the content of each specific photosynthetic pigment.

Leaf Chl. *a* = [12.7 (OD 663) − 2.69 (OD 645)] × *v*/1000 × *w* (3)

Leaf Chl. *b* = [22.9 (OD 645) − 4.68 (OD 663)] × *v*/1000 × *w* (4)

Leaf total Chl. = [20.2 (OD645) − 8.02(OD663)] × *v*/*w* × 1/1000 (5)

Leaf Car = A Car./Em 100% × 100
(6)

A carotenoid (µg/g FW) = ΔA480 + (0.114 × ΔA663) − (0.638 × ΔA645)
(7)

Emission = Em 100% = 2500
(8)

W = weight of the fresh leaf tissue (g)

V = volume of the extract (mL)

ΔA = absorbance at respective wavelength

### 2.9. Quantification of Leaf Total Phenolic Content (TPC)

For the quantification of TPC in fresh leaves the method of Julkenen-Titto [54] was employed. Fresh leaf material (0.05 g) was mashed in 80% acetone (5 mL) and centrifuged at 10,000× *g* at 25 °C for 10 min. The supernatant (100 µL) was then mixed with 1 mL Folin–Ciocalteau’s phenol reagent. To the resultant solution, dH_2_O (2 mL) and 20% Na_2_CO_3_ (5.0 mL) were added. After mixing well, the volume of the final triturate was raised to 10 mL with dH_2_O. The absorbance of the triturate was read at 750 nm using a UV–Visible spectrophotometer (IRMECO U2020) (GmbH, Geesthacht, Germany).

### 2.10. Quantification of Leaf Total Anthocyanin (T. Antho) Content

For the estimation of anthocyanin content, the method ascribed by Nakata et al. [55] was used with some modifications. Briefly, 50 mg fresh leaf material was homogenized in a mortar and pestle and mixed with 1 mL acidic methanol prepared by adding 1% HCl. The acidic methanol was prepared in an ice bath. After centrifugation of the homogenate at 14,000 rpm for 5 min at 25 °C, the absorbance of the supernatant was read at 530 and 657 nm. The following equation was then used for the quantification of the total anthocyanin content in leaves,

T. antho = (A530 − 0.25 × A657) × M^−1^ (9)

where A530 and A657 are the absorption at specific wavelengths, and M is the mass of fresh leaf used for the extraction (g).

### 2.11. Quantification of Leaf Ascorbic Acid (AsA) Content

For the quantification of AsA in fresh leaf, the well-known method of Mukherjee and Choudhuri [56] was used. Briefly, 0.25 g of fresh leaf material was homogenized in a mortar and pestle using 6% trichloroacetic acid solution (10 mL). The homogenate was then centrifuged at 10,000× *g* at room temperature for 10 min. The supernatant was then mixed with 2 mL of 2% acidic dinitrophenyl hydrazine solution. After adding 1 to 2 drops of 10% thiourea, the triturate was boiled in a water bath for 20 min. After cooling in an ice bath, 5 mL of 80% H_2_SO_4_ was added to the triturate. Then, the absorbance of the resultant mixture was read at 530 nm. For the quantification of AsA, known standards (50–300 ppm) were prepared from pure AsA and used following the same procedure used for the samples.

### 2.12. Quantification of Leaf Total Soluble Protein (TSP) and Activities of Antioxidant Enzymes

#### 2.12.1. Extraction of TSP and Enzymes

For the quantification of leaf TSP and activities of antioxidant enzymes, fully mature fresh leaves were collected; then, the fresh leaves (0.5 g) were homogenized in 10 mL chilled Na phosphate buffer having pH 7.8 in a cold mortar and pestle. The homogenate was then centrifuged at 4 °C for 20 min at 10,000× *g*. The supernatant was used for quantifying TSP and antioxidant enzymes activities.

#### 2.12.2. Estimation of TSP

For the quantification of TSP, the method ascribed by Bradford [57] was used. Briefly, 2 mL of Bradford reagent was added to 100 µL buffer extract, mixed well, and the absorbance was then spectrophotometrically read at 595 nm. The quantification was made using a standard curve drawn from the pure standards (200–1600 ppm) prepared from analytical grade bovine serum albumin (BSA).

#### 2.12.3. Determination of the Activity of Superoxide Dismutase (SOD)

For the estimation of SOD activity, the method of Giannopolitis and Ries [58] was used. Briefly, a mixture comprised 50 mM phosphate buffer (pH 7.8), 1.3 μM riboflavin, 50 μM NBT, 75 nM EDTA, 13 mM methionine, and 50 μL enzymatic extract was prepared. The finally prepared mixture was kept under a fluorescent light source of 20 W for 15 min. The reduction inhibition of NBT was measured at 560 nm. The activity in samples was estimated as unit/mg protein using the content of TSP.

#### 2.12.4. Estimation of Peroxidase (POD) Activity

The activity of POD in leaf buffer extract was determined following the method ascribed by Chance and Maehly [59]. Oxidation of the guaiacol in reaction mixture was taken as the basic mechanism for the estimation of POD activity. One milliliter of the reaction mixture was prepared using 50 mM phosphate buffer, 100 μL leaf phosphate buffer extract, 40 mM H_2_O_2_, and 20 mM guaiacol. The change in color of reaction mixture due to oxidation of guaiacol was spectrophotometrically read at 470 nm with an interval of 20 sec in a time scan manner. Final activity was quantified as units/mg protein.

#### 2.12.5. Determination of Catalase (CAT) and Ascorbte Peroxidase (APX) Activity

For the determination of CAT activity in leaf buffer extract, the method ascribed by Chance and Maehly [59] was followed. The estimation was based on the disappearance of H_2_O_2_ as basic phenomenon in reaction mixture measured as decrease in absorbance at 240 nm. The reaction mixture comprised 100 mL of extract, 1 mL dH_2_O, and 1.9 mL H_2_O_2_. However, the method given by Asada and Takahashi [60] was used to measure the activity of APX. The decrease in absorbance of reaction mixture was read spectrophotometrically at 290 nm and expressed as units mg^−1^ protein.

### 2.13. Estimation of Total Free Amino Acid (FAA) Content

For the quantification of FAA, the method given by Hamilton and Van-slyke [61] was used. Briefly, the reaction mixture was prepared by adding 1 mL of the extract and 1 mL of 1% pyridine and 2% ninhydrin. After heating the mixture for 30 min at 95 °C in water bath, the final volume was made up to 50 mL at room temperature and the absorbance of triturate was read at 570 nm. The content of FAA was quantified using the following equation.

Total FAA = Abs × V × DF/wt of sample × 1000
(10)

### 2.14. Quantification of Leaf Reducing Sugars (RS)

For the quantification of leaf reducing sugars, the method given by Wood and Bhat [62] was used. Briefly, 0.5 g of fresh leaves was homogenized in methanol (80%) followed by centrifugation for 20 min at 10,000× *g* at room temperature. Then, the reaction mixture was prepared by adding 1 mL in 4 mL of the DNS followed by heating for 5 min in a water bath at 95 °C. The mixture was then cooled immediately in chilled water and incubated at 25 °C. The absorbance of the finally prepared mixture was read at 540 nm.

### 2.15. Quantification of Total Soluble Sugars (TSS)

For the quantification of leaf total soluble sugars, 0.1 mL of the methanolic extract was mixed with 3 mL of antheron reagent followed by heating at 95 °C for 10 min. After cooling in chilled water, the mixture was incubated at room temperature for 30 min. The absorbance of the triturate was read at 625 nm using a spectrophotometer. The final quantification was made using a standard curve prepared from pure standards (200–1000 ppm).

### 2.16. Quantification of Non-Reducing Sugars (NRS)

The NRS were estimated using the following formula.

NRS = TSS − RS
(11)

### 2.17. Quantification of Leaf Malondialdehyde (MDA) Content

For the quantification of MDA content in fresh leaves, the Cakmak and Horst [63] method was used. Briefly, 1 g of fresh leaf material was homogenized in TCA (10%) solution, followed by centrifugation at 10,000× *g* for 20 min at room temperature. Then, the reaction mixture was prepared by adding 0.5 mL supernatant in 3 mL of thiobarbituric acid (TBA) solution, prepared in 20% TCA followed by heating for 50 min at 95 °C in a water bath. The mixture was then cooled in chilled water. After centrifugation, the absorbance of colored part was read at 532 and 600 nm. The quantification of MDA content was made using the formula

MDA (nmol) = Δ (A 532 nm − A 600 nm)/1.56 × 105.
(12)

The absorption coefficient for the calculation of MDA is 156 mmol^−1^ cm^−1^.

### 2.18. Quantification of Leaf H_2_O_2_ Content

For the determination of leaf H_2_O_2_ content, the method ascribed by Velikova et al. [64] was used. Briefly, 0.5 g fresh leaf was homogenized in 10 mL of 6% TCA solution. After centrifugation at 10,000× *g* for 10 min, 0.1 mL of the obtained supernatant was mixed with 1 mL saturated solution of KI. After mixing, the absorbance was read at 390 nm and the quantification was made by drawing a standard curve prepared from a range of pure standards.

### 2.19. Quantification of Amino Acids

#### 2.19.1. Quantification of Leaf Glycine Betain (GB) Content

The method ascribed by Grieve and Grattan [65] was used for the quantification of GB content in the leaves. Chopped leaf material (1 g) was extracted in ddH_2_O, kept overnight, and then filtered through Watman No. 1 filter paper. One milliliter of filtrate was mixed with 1 mL HCl (2 N). Then, 0.5 mL of the mixture was mixed with 0.2 mL solution of potassium iodide. To the mixture, 20 mL cooled dichloromethane and 2 mL of chilled ddH_2_O were added, followed by shaking along with a continuous flowing of air. After settling well, the lower phase was separated and the absorbance was read at 365 nm using a spectrophotometer. The quantification of GB in samples was done by using a standard curve prepared from known standards (5–25 ppm) following the same procedure as for leaf samples.

#### 2.19.2. Quantification of Proline (Pro) Content in Fresh Leaves

For the quantification of proline content in fresh leaves, the method given by Bates et al. [66] was used. Fresh leaf material (0.1 g) was homogenized in 3% solution sulfosalicylic acid (5 mL) using a mortar and pestle, followed by filtration. Then, 100 µL of the filtered extract were mixed with 20 mL of phosphoric acid (6M). The triturate was then mixed with 2 mL of glacial acetic acid and 2 mL of acidic ninhydrin followed by heating at 95 °C in a water bath for 1 h. The mixture was then cooled immediately by using chilling water and 4 mL of the toluene were added to the mixture. The colored phase was then separated and the absorbance was read at 520 nm. Concentration of proline was determined using standard curve prepared from a range of pure standards (5–25 ppm) following the same procedure used for the samples. The following equation was used to measure the quantity of proline in the samples.

Proline µmol g^−1^ Fw = mL of toluene/115 g × µg proline mL^−1^)/sample (g) (13)

#### 2.19.3. Quantification of Aspartate (Asp) Content in Leaves

The quantification of the aspartic acid content in fresh leaves was done from the buffer extracted solution using the method of Pfleiderer et al. [67]. Briefly, the reaction mixture was prepared by adding 0.1 mL of DPNH solution in 0.1 mL of extract, followed by the addition of 0.03 mL of transaminase (30 units), 2.7 mL of phosphate buffer, and 0.02 mL of malic dehydrogenase (600 units). The absorbance of the mixture was read at 340 nm and then 0.05 mL of α-ketoglutarate was added to the mixture and the decrease in absorbance was read for 15 min. Then, the aspartic acid concentration in samples was estimated using the following equation given below.

(14) $$C=\frac{E\times 133}{6.22\times 10^{6}}\;\left(g\;\mathrm{aspartic}\;\mathrm{acid}/\mathrm{mL}\right)$$

6.22 × 10^6^ molar extinction coefficient of DPNH at 340 nm, 133 = molecular weight of aspartic acid, where E = extra plotted extinction decrease.

#### 2.19.4. Quantification of Glutamate (Glu) Content in Leaves

The method of Beutler and Michal [68] was used for the estimation of glutamate in buffer-extracted solution. The formation of 2-oxoglutarate by the oxidative deamination was the basic mechanism in the presence of glutamate dehydrogenase and NAD. In the presence of NADH, the formation of iodonitrotetrazolium chloride (INT) with diaphorase takes place by oxidative process and the absorbance of INT was read at 492 nm. Briefly, the reaction mixture was prepared by adding diaphorase (0.14 i.u./mL), NAD (0.38 mM), INT (0.068 mM), 500 μL sample triethanolamine (57 mM), GDH (14i.u./mL), and potassium phosphate (14 mM, pH 8.6). The absorbance of the finally prepared mixture was read at 492 nm using spectrophotometer against a reagent blank.

#### 2.19.5. Quantification of Methionine (Meth) and Lysine (Lys) Contents in Leaves

For the estimation of Leaf Meth and Lys content, the method ascribed by Losak et al. [69] was used. Leaf samples (1 g) were homogenized well in 10 mL HCl (0.1%) using a mortar and pestle. After centrifugation at 10,000× *g*, the supernatant was divided in two parts and one part was mixed with phosphate buffer (50 mM), ninhydrin solution, and 50% glycerol in order to extract lys, followed by heating in a water bath at 95° and the absorption of the mixture was read at 570 nm. For the extraction of methionine, the other half part of the supernatant was mixed with glycine dihydrate (50%), sodium nitroferricyanide dihydrate (0.1%), NaOH (5N), and HCl (1:1), and the absorbance of the triturate was read at 510 nm.

### 2.20. Quantification of Reduced Glutathione (GSH) and Oxidized Glutathione (GSSG)

The quantification of GSH and GSSG was assayed using the method as ascribed by Griffith [70]. The fresh leaf material (0.5 g) was homogenized in 2 mL of 0.1 M HCl having 1 mM EDTA using a pestle and mortar. The homogenate was centrifuged at 15,000× *g* at 4 °C for 20 min. The reaction mixture was prepared by adding 100 μL DTNB (6.0 mM), 500 μL NADPH (0.3 mM), 200 μL extract, and 200 μL phosphate buffer (125 μM) having 6.3 mM EDTA (pH 7.5). The absorbance of triturate was read spectrophotometrically at 412 nm.

### 2.21. Determination of Yield Attributes

For the estimation of yield attributes, the plants were harvested at maturity and their capitula were separated. The yield related parameters such as 100 seed weight, seed yield per capitulum, and capitulum’s diameter were determined manually.

### 2.22. Quantification of Minerals in Shoots and Roots

For the quantification of minerals in shoot and root tissues, the dry samples were ground to powdered and digested following the well-known digestion method as ascribed by Wolf [71] using the sulfuric acid. The cations such as Ca^+^, K^+^, Mg^2+^, and Fe^2+^ were estimated using the atomic absorption spectrophotometer (AAS) (Hitachi. Model 7JO-8024, Tokyo, Japan). Quantification of P in the digests was spectrophotometrically done using Barton’s reagent method. However, the quantification of N content from the digested material was done using the method described by Bremner and Keeney [72].

### 2.23. Statistical Analysis

To determine the significant differences of the treatments, the obtained data for different studied attributes were analyzed statistically using Co-STAT version 6.3 (developed by Cohort Software Berkley, California, CA, USA). The significant difference among mean values of different treatments and least significance difference (LSD) test at 5% level of significance were assayed. Correlations and PCA analyses of the studied attributes were computed using XLSTAT software and the significance among the generated values against each attribute was determined using the Spearman’s correlation Table.

## 3. Results

### 3.1. Growth and Yield Attributes

A significant reduction in the studied growth attributes of sunflower plants such as SFW, RFW, SDW, SFW, SL, and RL was recoded due to water stress. Significant improvements were recorded in these growth attributes of water-stressed sunflower plants due to foliar fertigation with both levels of Fe-Asp and FeSO_4_. Foliar fertigation of both levels of Fe-Asp was found more effective for improvement of all studied growth attributes of water-stressed sunflower plants, in comparison with both levels of FeSO_4_. However, SL 0.5% level of Fe-Asp was comparatively more effective (Table 1).

Limited water supply imposed significantly adverse effects on different studied yield parameters including the capitulum diameter, number of achenes, achene weight per capitulum, and 100 achene weight of sunflower plants. Foliar fertigation with different levels of FeSO_4_ and Fe-Asp significantly improved all the studied yield attributes. Comparatively, a greater improvement in the yield attributes was found due to fertigation with Fe-Asp. However, in the case of capitulum diameter, the lower level of FeSO_4_ and Fe-Asp was found more effective. In the case of number of achenes per capitulum, the 0.5% level from both Fe sources was found more effective. However, in case of achene weight per capitulum, the 0.5% level of Fe-Asp was most effective (Table 1).

Imposition of water stress significantly increased the accumulation of GSSG and GSG in sunflower plants. This accumulation of GSG and GSSG was further increased due to foliar fertigation with different levels of Fe-Asp and FeSO_4_ both under water stressed and non-stressed conditions. Comparatively, this improvement was more effective due to the foliar fertigation with Fe-asp in comparison with FeSO_4_ (Table 1).

### 3.2. Leaf Relative Water Content, Photosynthetic Pigments, Carotenoids, and Anthocyanin Contents

Data presented in Table 2 for leaf total Chl. Chl. *a*, Chl. *b*, and Chl. *a*/*b* shows that limited irrigation increased significantly the content of leaf total Chl., Chl. *A*, and Chl. *b* but a decrease was found in Chl. *a*/*b*, indicating that under water deficit conditions, more improvement was found in Chl. *b* as compared with Chl. *a*. Foliar fertigation with different levels of FeSO_4_ and Fe-Asp at vegetative stage significantly increased the contents of leaf photosynthetic pigments in water-stressed sunflower plants. Comparatively, more improvement in these photosynthetic pigments was due to foliar fertigation of Fe-Asp_._ Furthermore, this high increase in leaf photosynthetic pigments due to foliar-supplied Fe-Asp was the same at both levels. However, in case of leaf Ch. *a*/*b*, the more increase was found at higher levels (0.5%) from both Fe sources.

Significant increases in leaf carotenoids and total anthocyanin contents were recorded in sunflower plants when grown under limited irrigation (Table 2). Foliar fertigation with different levels of Fe-Asp and FeSO4 further significantly increased the leaf carotenoids and anthocyanin contents in sunflower plants grown in water deficit conditions. Furthermore, the foliar fertigation of Fe-Asp was found more effective in further increasing the leaf carotenoids and anthocyanin contents.

Leaf relative water content of water-stressed sunflower plants significantly decreased. Foliary-supplied FeSO_4_ and Fe-Asp significantly enhanced the LRWC of water-stressed sunflower plants. This improvement in LRWC was comparatively more in sunflower plants foliary-fertigated with both levels of Fe-Asp (Table 2).

### 3.3. Contents of Different Amino Acids

**Figure 2:**
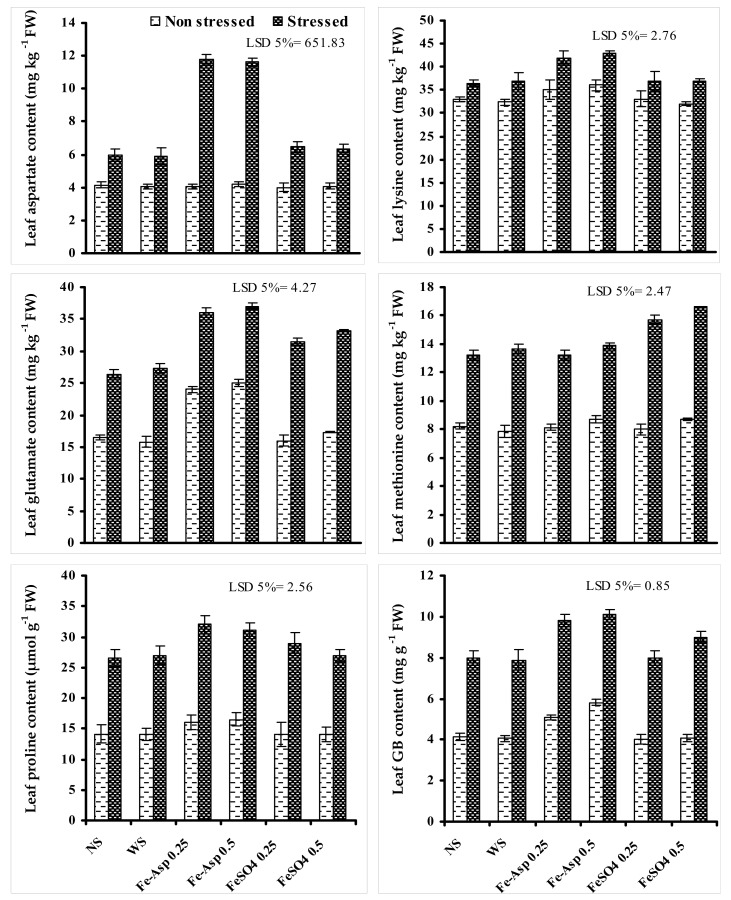
Influences of foliary fertigated Fe-Asp and FeSO_4_ on the contents of different leaf amino acids of sunflower (*Halianthus annus* L.) plants when grown under limited irrigation (mean ± SE; *n* = 3).

A significant increase was found in leaf Asp, lysine, glutamate, methionine proline, and GB contents of sunflower plants under limited irrigation. Fertigation with both levels of Fe-Asp only further significantly increased the accumulation of the leaf Asp, lysine, glutamate, proline, and GB of sunflower plants grown under water stress except to that of the glutamate content that was also improved, as when foliary supplied both levels of FeSO_4_ under water stress and 0.5% level of FeSO_4_ were more effective. However, only the foliar-applied FeSO_4_ enhanced the leaf methionine content of water stressed sunflower plants and a slightly more increase was recorded at a higher level of FeSO_4_ (Figure 2).

### 3.4. LRMP, MDA, FAA, H_2_O_2_, and Activities of Different Antioxidant Enzymes

**Figure 3:**
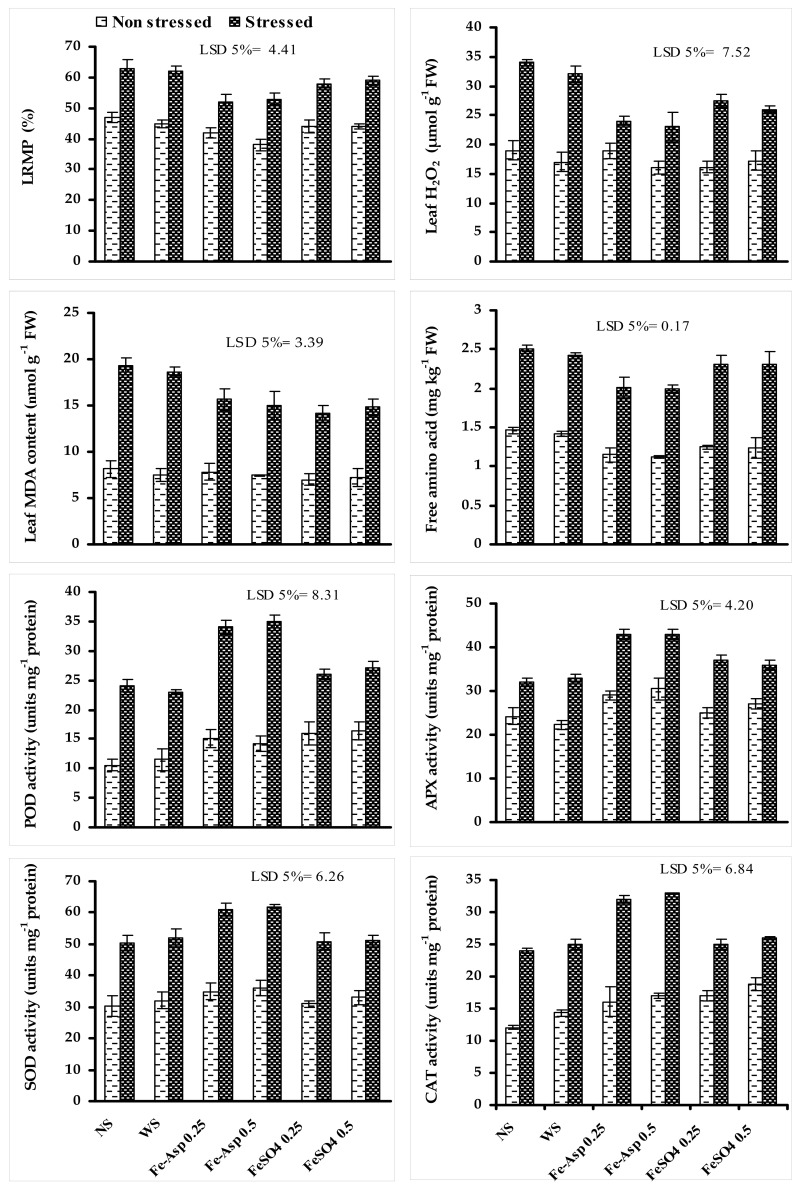
Influences of foliary fertigated Fe-Asp and FeSO_4_ on leaf LRMP; contents of H_2_O_2_, MDA, and FAA; and activities of SOD, POD, CAT, and APX of sunflower (*Halianthus annuus* L.) plants when grown under limited irrigation (mean ± SE; *n* = 3).

The data presented in Figure 3 show that LRMP and the contents of FAA, MDA, and H_2_O_2_ of sunflower plants significantly increased when grown under limited water supply. Fertigation with both levels of FeSO_4_ and Fe-Asp significantly reduced the adverse impacts of water shortage on LRMP, and decreased the contents of H_2_O_2_, MDA, and FAA of water-stressed sunflower plants. A greater decrease in LRMP and the contents of H_2_O_2_ and FAA was recorded in water-stressed sunflower plants fertigated with both levels of Fe-Asp (Figure 3).

Water stress significantly increased the leaf CAT, APX, POD, and SOD activities of sunflower plants (Figure 3). These activities of CAT, APX, POD, and SOD of sunflower plants under limited irrigation were further increased significantly due foliar fertigation with both levels of FeSO4 and Fe-Asp. Both levels of Fe-Asp were found to be more effective at increasing the activities of POD, APX, SOD, and CAT under water stressed condition as compared with both levels of FeSO_4_. This high increase in enzymes activities was the same at both levels of Fe-Asp. This increase in activities of enzymes due to foliar-sprayed Fe-Asp and FeSO_4_ was also found in non-stressed sunflower plants, but the improvement was dependent on the treatment type and level (Figure 3).

### 3.5. Contents of AsA, Phenolics, Flavonoids, Total Soluble Proteins, Total Soluble Sugars, Reducing Sugars, and Non-Reducing Sugars

**Figure 4:**
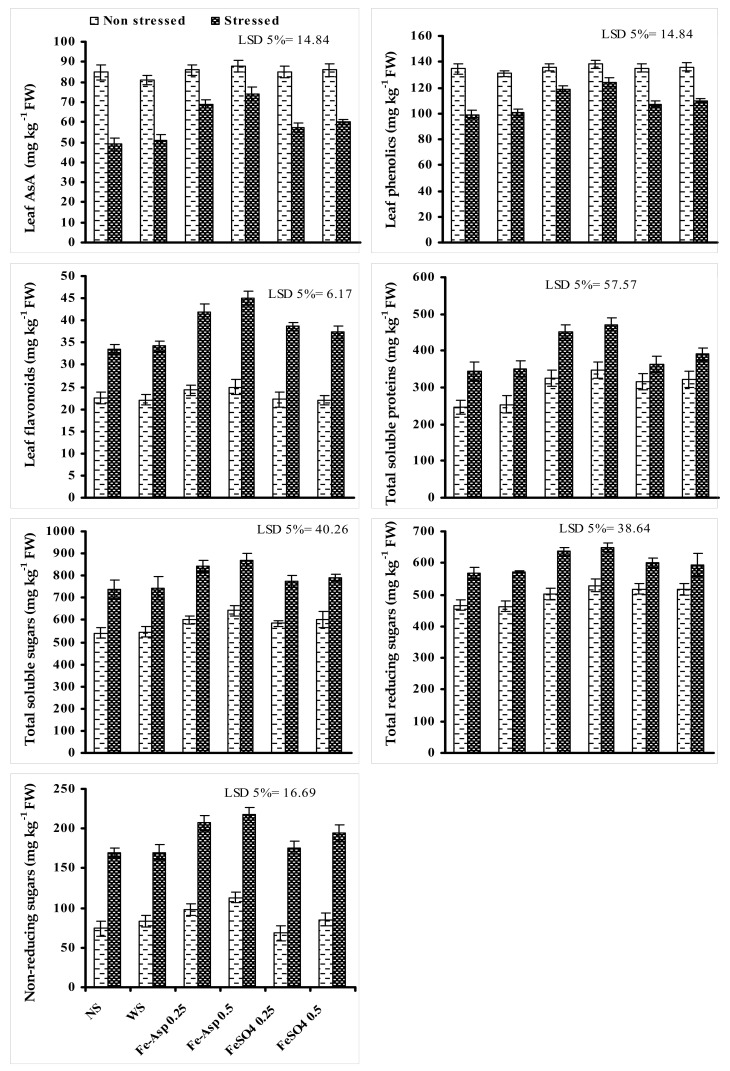
Influences of foliary fertigated Fe-Asp and FeSO_4_ on contents of leaf AsA, TSP, TSS, RS, and NRS of sunflower (*Halianthus annuus* L.) plants when grown under limited irrigation (mean ± SE; *n* = 3).

Leaf AsA and total phenolic contents decreased due to the limited irrigation. Fertigation of FeSO_4_ and Fe-Asp with foliar spray was found to be effective in increasing the leaf AsA and total phenolic content of water stressed sunflower plants and comparatively Fe-Asp fertigation was found more effective at both levels. Leaf total flavonoids and TSP contents increased in sunflower plants when grown under reduced irrigation. A further significant increase in TSP and total flavonoids was found in plants fertigated with different levels of FeSO_4_ and Fe-Asp under both water-stressed levels, and a greater increase was found in plants foliary fertigated with Fe-Asp at both levels. Comparatively, F-Asp at a higher level (0.5%) was slightly more effective as compared with the lower level (Figure 4).

Water stress significantly reduced the contents of leaf TSS, non-reducing sugars, and reducing sugars of sunflower plants. The content of TSS, reducing sugars, and non-reducing sugars was further significantly increased in water-stressed sunflower plants due to the foliary supplied Fe-Asp and FeSO_4_, and both levels of Fe-Asp were found comparatively more effective in increasing the content of TSS, reducing sugars, and non-reducing sugars. In the case of non-reducing sugars, such an increase was greater at a higher level (0.5%) of both Fe-Asp and FeSO_4_. Furthermore, this increase in leaf TSS, non-reducing sugars, and reducing sugars was also found in non-stressed sunflower plants (Figure 4).

### 3.6. Contents of Different Minerals in Shoot and Root

Uptake of nutrients such as N, P, K, Ca, and Mg in roots and shoots decreased significantly in sunflower plants when grown under reduced irrigation (Table 3). Significant improvement in the uptake of N, P, K, Ca, and Mg in roots and shoots was recorded in sunflower plants foliary fertigated with both levels of Fe-Asp and FeSO4 under non-stressed and water deficit conditions. Comparatively, a greater increase in the uptake of the nutrients in roots and shoots was found in sunflower plants grown under stressed and non-stressed conditions and foliar-applied with both levels of Fe-Asp than FeSO_4_, except for that of shoot K where the higher level (0.5%) of Fe-Asp was more effective. Furthermore, in case of root Ca, fertigation with higher level of Fe-Asp was found better under water deficit conditions.

A significant reduction in root and shoot Fe content was also found in sunflower plants due to the water stress. A significant increase in root and shoot Fe content was found in sunflower plants when fertigated with Fe-Asp and FeSO_4_, and comparatively more increase in root and shoot Fe content was found in plants fertigated with both levels of Fe-Asp as compared with FeSO_4_ under both water regimes. Furthermore, the higher level (0.5%) of Fe-Asp was found more effective in improving the root and shoot Fe content under both water stress levels. However, in case of foliary applied FeSO_4_, both levels were found equally effective in improving root and shoot Fe (Table 3).

### 3.7. Correlations among the Attributes of Water Stressed Sunflower Plants

**Figure 5:**
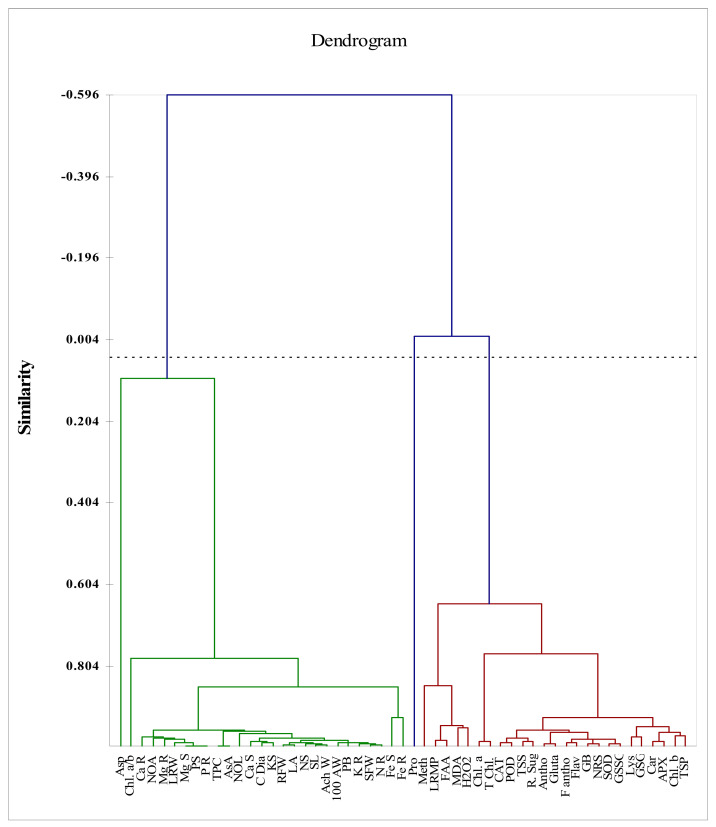
Correlations among the studied attributes of water stressed sunflower plants.

The correlations among plant growth yield attributes with all other attributes are presented in Figure 5 and Table 4. All study attributes have been arranged in two groups, as presented in Figure in dendrogram in circles. The growth and yield attributes such as SFW, RFW, LA, PB, 100AW, AW, CDia, and NOA have a strong positive correlation with root and shoot nutrients such as N, P, K, Ca, and Mg. However, these growth attributes, yield attributes, and nutrients in roots and shoots have significant positive correlation with Chl. *a*/*b* ratio, but a non-significant correlation with asp content. The other studied attributes such as antioxidative defense mechanism, photosynthetic pigments, membrane permeability, H_2_O_2_ and amino acids, including Lys, Glu, GB, and Meth, have strong positive significant correlation with each other. The circles showing the parameters in Figure 5 have negative correlations among each other. Plant biomass has positive significant correlation with shoot and root K (0.959 *** and 0.993 ***), Ca (0.972 *** and 0.954 ***), N (0.987 *** and 0.992 ***), P (0.981 *** and 0.981 ***) and Mg (0.980 *** and 0.985 ***), 100 AW (0.990 ***), AW (0.987 ***). Plant biomass also has a positive correlation with TPC (0.960 ***), AsA (0.960 ***). Shoot and root Fe contents also showed a strong positive and significant correlation with all other attributes in circle.

## 4. Discussion

Under limited water supply, the uptake of nutrients represents an important issue that adversely affects crop production. Fe deficiency, along with other nutrients under limited water supply, is also a problem that negatively affects the crop yield and nutritional quality [22]. Different fertilizers are being employed to improve plant Fe internal levels and have been found effective to some extent. However, studies reveal that these fertilizers also impose negative impacts in some instances and are not effective as compared with chelated compounds [21,73]. This negative or lesser effectiveness is attributed to the greater chances of the oxidation of metal part or due to the partner cation, and same is with the exogenous application of FeSO_4_. Furthermore, the mobility and translocation of micronutrient is also not up to the mark when applied in chelated form. In mineral nutrition/fertilization the interest is increasing in ecofriendly chelated compounds due to better uptake, translocation and beneficial effects [31].

### 4.1. Plant Growth and Seed Yield

In the present study, the limited irrigation adversely affected growth and yield of sunflower plants. Previous studies revealed that the final production depends on the biomass status of the plants [8]. Similar is in the present findings where exogenous application of FeSO_4_ and Fe-Asp, when applied foliary, significantly improved the plant growth and yield, being more improvement in sunflower plants foliary fertigated with Fe-Asp in comparison to FeSO_4_. This might be due to the more mobility and translocation of Fe in terms of Fe-Asp [31] as well as due to the lower oxidation reaction chances being organic in nature with a chelation of Asp amino acid. Additionally, the counterpart Asp might also play a significant role in mediating sunflower tolerance to water stress. Moreover, the Fe content in root and shoot was significantly improved in sunflower plants and more increase in Fe content was recorded in plants fertigated with Fe-Asp. Furthermore, the amino acids (aspartate, glutamate, lysine, and methionine) belonging to Asp family were also improved significantly along with the content of Asp that correlates well with better biomass and seed yield of water-stressed sunflower plants. Studies reveal that Fe has a significant role in stress tolerance induction of many plant species when applied exogenously [28,29]. For example, it was reported by Ramezani et al. [28] that foliary-supplied Fe has a significant impact on nutrient uptake and yield of water-stressed alfalfa plants. They found that foliary applied of Fe as FeSO_4_ considerably increased the nutrient acquisition as well as yield of water stressed alfalafa plants. Furthermore, foliary applied FeSO_4_ improved the stress tolerance ability of *Calendula officinalis* L. plants, resulting in better growth and yield [29]. Foliary applied aspartate also improved growth and stress tolerance of rice plants due to improved photosynthetic efficiency and better nutrients uptake [32]. In another study conducted by Akladious and Abbas [74], it was found that foliary applied aspartate significantly improved the plant growth with an increase in amino acid biosynthesis along with an improvement in antioxidative defense mechanism and plant stress tolerance.

### 4.2. Role of Leaf Photosynthetic Pigments

The better performance in terms of yield and growth/biomass production depends on the performance of different physio-biochemical attributes, especially the performance of leaf photosynthetic pigments that play significant role in net photosynthesis, and assimilation process by capturing the sunlight. In the present study, leaf photosynthetic pigments of sunflower plants were significantly improved due to the fertigation with FeSO_4_ and Fe-Asp, being more in plants fertigated with Fe-Asp. Under water stress, process of electron transport disturbs due to the deficiency of Fe as well as the oxidative damages to chloroplastic photosynthetic membranes by reactive oxygen species [75] that might also have been found in terms of increased lipid peroxidation to water-stressed sunflower plants. Exogenously applied Fe in both forms (Fe-Asp and FeSO_4_) was found to be effective in ameliorating the adverse impacts of imposed drought stress on the biosynthesis of leaf photosynthetic pigments by decreasing membrane lipid peroxidation that might have improved the leaf photosynthetic efficiency with better growth and yield of water stressed sunflower plants. Previous studies have also reported that the exogenous Fe application improved the stress tolerance by boosting up the plant photosynthetic efficiency of maize [76], sunflower [77] and *W. murcott* mandarin [78]. Furthermore, it was reported by Sadak et al. [78] that foliary-applied Asp increased the stress tolerance of *Vicia faba* plants associated with improved accumulation of phenolic content, proline, amino acids, total soluble sugars, polyamines, and IAA [79]. In rice plants (20 mg kg^−1^), foliar spray of Asp improved the leaf photosynthetic pigments that lead to better plant growth under stressful environment [32]. Therefore, the better performance of sunflower plants to tolerate the drought when fertigated with Fe-Asp might be due to the additional role of Asp in parallel with improved Fe content.

### 4.3. Maintenance of Plant Water Relations and Osmotic Adjustment

Better maintenance of plant water relations under stressful environment especially under limited water supply is of prime importance for stress tolerance that has direct effects on the plant growth and production by disturbing the plant photosynthetic efficiency, assimilation, and the nutrient uptake [80]. In order to cope with such perturbations, different important mechanisms have been developed to maintain their cellular water content through osmotic adjustment by the accumulation of different osmotia that results in more cellular negative osmotic potential as a result better water uptake from soil under limited water supply [9,81]. The present study revealed a significant improvement in LRWC and nutrient uptake due to the foliary-applied Fe-Asp and FeSO_4_ that correlate well with better growth and production. Similarly, foliar application of Fe improved plant water relations of water stressed *Phaseolus vulgaris* L. plants that resulted in improved yield [82]. The improvement in cellular water relation was more pronounced in case of Fe-Asp as compared with FeSO_4_. This might be due to the direct involvement of Asp in osmotic adjustment as well as the high accumulation of different amino acids and enhanced nutrient uptake in case of Fe-Asp. The osmolytes that have significant role in cellular osmotic adjustment include amino acids, sugars as well as nutrient assimilation [9,33,83,84]. Similarly, it was found that in rice plants, foliar spray of Asp improved the nutrient uptake and cellular water content under stressful conditions [32]. In *Moringa olefera*, it was found that Asp accumulation significantly improved the cellular amino acid accumulation, resulting in better cellular water relations and improved photosynthetic efficiency [85]. The findings of the present study correlate well with the outcomes of previous studies because the foliary-applid Fe-Asp significantly improved the contents of different amino acids such as methionine, glutamate, proline, lysine, GB, as well as their own contents, indicating their significant role in induction of stress tolerance by improving cellular water relations [33]. Among those, proline and GB are well known and are considered important [9,86]. The better cellular amino acid accumulation and mineral nutrients correspond well to improved biomass production and better seed yield in association with the maintenance of better water relation. Furthermore, previous studies revealed that plants have the ability to translocate foliary supplied organic compounds to different tissues which enhances the accumulation of organic compounds [8]. These organic compounds also boost up their own biosynthesis by regulating their metabolism.

### 4.4. Improvement in Antioxidative Defense Mechanism

Oxidative stress tolerance (scavenging of excessive production of ROS) is another mechanism that has a necessary role in tolerance of plant to stressful environment by reducing the damaging effects of ROS on cellular membranes through the improvement of antioxidant enzymes activities and enhancement of the biosynthesis of non-enzymatic antioxidant [87,88,89]. The enzymatic antioxidants include the CAT, POD, SOD, APX, and the non-enzymatic ones are the AsA, Toc, Car, TPC, TFC, and anthocyanins. These antioxidative components scavenge the over-produced ROS [90,91,92]. In present study, the exogenously applied Fe-Asp and FeSO_4_ significantly improved the antioxidative defense mechanism of sunflower plants that can be accessed from the lower accumulation of MDA and H_2_O_2_ in sunflower plants, accompanied with a better performance of drought stressed sunflower plants with better growth and yield. The foliary-applied Fe sources improved the oxidative stress tolerance of sunflower plants when grown under limited water supply by increasing the activities of antioxidative enzymes such as APX, POD, SOD CAT, and the accumulation of non-enzymatic components such as TPC, TFC, AsA, and tocopherols. This improvement in the antioxidant components is due to the foliar application of FeSO_4_ and Fe-Asp that correlate well with the lowered MDA and H_2_O_2_ contents, showing less damage to cellular membranes [8,93,94].

## 5. Conclusions

In conclusion, foliar applied Fe-Asp and FeSO_4_ were found effective in ameliorating the adverse effects of water stress on growth and yield of sunflower plants, but this improvement was higher in case of Fe-Asp when applied as a foliar spray. The considerable amelioration might be due to the effective involvement of foliarly applied Fe-Asp in improving the biosynthesis of photosynthetic pigments for better photosynthesis, enhanced maintenance of plant water relations through amino acid metabolism, and efficient improvement in nutrient acquisition and antioxidative defense mechanisms. Overall, the recommendations at farmer end goes in the favor for the use of Fe-Asp under limited water supply as well as under normal irrigation to obtain better seed yield. Moreover, the exogenous use of Fe-Asp will be favored due to its eco-friendly nature in comparison with FeSO_4_.

## Figures and Tables

**Table 1:** Influences of foliary fertigated Fe-Asp and FeSO_4_ on different growth and yield parameters of sunflower (*Halianthus annus* L.) plants when grown under limited irrigation (mean ± SE; *n* = 3).

**Treatment**	**SFW (g Plant^−1^)**		**RFW (g Plant^−1^)**		**PDW (g Plant^−1^)**		**SL (cm)**		**LA (cm^2^)**		**Achene wt./Capitulum (g)**	
	**Non-Stressed**	**Water-Stressed**	**Non-Stressed**	**Water-Stressed**	**Non-Stressed**	**Water-Stressed**	**Non-Stressed**	**Water-Stressed**	**Non-Stressed**	**Water-Stressed**	**Non-Stressed**	**Water-Stressed**
**NS**	536 ± 9.82 ^c,d^	392 ± 34.0 ^c^	99 ± 3.46 ^a,b^	65 ± 4.33 ^b^	170 ± 1.53 ^b^	125 ± 5.40 ^b^	50.5 ± 0.87 ^e^	41 ± 1.44 ^d^	1326 ± 26 ^c^	825 ± 64 ^b^	0.61 ± 0.02 ^c^	0.41 ± 0.02 ^d^
**WS**	527 ± 14.0 ^d^	385 ± 14.0 ^c^	101 ± 4.62 ^a,b^	68 ± 6.64 ^b^	168 ± 6.52 ^b^	127 ± 6.60 ^b^	51.5 ± 0.87 ^d^	42 ± 1.04 ^c^	1354 ± 30 ^b,c^	856 ± 22 ^b^	0.64 ± 0.01 ^b^	0.44 ± 0.01 ^c,b^
**Fe-Asp 0.25%**	572 ± 12.0 ^a,b^	457 ± 5.20 ^a^	103 ± 1.23 ^a,b^	76 ± 1.73 ^a^	181 ± 5.31 ^a^	147 ± 8.50 ^a^	54.0 ± 1.15 ^b^	44 ± 0.58 ^b^	1450 ± 11 ^a^	981 ± 45 ^a^	0.68 ± 0.02 ^a^	0.49 ± 0.02 ^b^
**Fe-Asp 0.5%**	584 ± 7.51 ^a^	468 ± 31.4 ^a^	105 ± 2.31 ^a^	78 ± 2.88 ^a^	186 ± 3.75 ^a^	145 ± 1.76 ^a^	55.0 ± 1.15 ^a^	45 ± 2.02 ^a^	1432 ± 32 ^a^	988 ± 33 ^a^	0.69 ± 0.04 ^a^	0.52 ± 0.02 ^a^
**FeSO4 0.25%**	544 ± 7.22 ^c,d^	417 ± 13.0 ^b^	98 ± 1.16 ^b^	68 ± 1.73 ^b^	165 ± 7.25 ^b^	132 ± 2.02 ^b^	51.0 ± 0.29 ^d^	42 ± 1.15 ^c^	1356 ± 60 ^b,c^	861 ± 54 ^b^	0.63 ± 0.02 ^b,c^	0.44 ± 0.02 ^c^
**FeSO4 0.5%**	550 ± 8.66 ^b,c^	421 ± 14.4 ^b^	99 ± 1.73 ^a,b^	67 ± 2.63 ^b^	168 ± 8.11 ^b^	133 ± 7.80 ^b^	52.0 ± 0.29 ^c^	42 ± 0.58 ^c^	1375 ± 66 ^b^	863 ± 34 ^b^	0.64 ± 0.03 ^b^	0.45 ± 0.01 ^c^
**LSD**	22.2		5.48		8.96		0.74		45.84		0.41	
**Treatment**	**Capitulum dia. (cm)**		**Achenes per capitulum**		**100 grain wt. (g)**		**GSSG**		**GSG**			
	**Non-Stressed**	**Water-Stressed**	**Non-Stressed**	**Water-Stressed**	**Non-Stressed**	**Water-Stressed**	**Non-Stressed**	**Water-Stressed**	**Non-Stressed**	**Water-Stressed**		
**NS**	13.33 ± 1.18 ^b^	10.00 ± 0.99 ^c^	270 ± 13 ^b,a^	237 ± 13 ^d^	0.32 ± 0.011 ^a,b^	0.18 ± 0.01 ^b^	6.23 ± 0.41 ^c^	14.33 ± 0.88 ^b^	0.21 ± 0.011 ^b^	0.41 ± 0.015 ^b^		
**WS**	13.87 ± 1.20 ^a,b^	10.33 ± 1.19 ^b,c^	280 ± 14 ^b,c^	238 ± 16 ^d^	0.31 ± 0.012 ^b^	0.19 ± 0.02 ^b^	6.89 ± 0.48 ^b,c^	14.63 ± 0.98 ^b^	0.22 ± 0.010 ^b^	0.43 ± 0.013 ^b^		
**Fe-Asp 0.25%**	14.26 ± 0.87 ^a^	11.85 ± 0.98 ^a^	300 ± 14 ^a^	263 ± 10 ^b^	0.35 ± 0.010 ^a^	0.23 ± 0.02 ^a^	7.45 ± 0.23 ^a,b^	17.62 ± 1.10 ^a^	0.33 ± 0.011 ^a^	0.56 ± 0.011 ^a^		
**Fe-Asp 0.5%**	14.63 ± 0.89 ^a^	11.03 ± 0.63 ^a,b^	305 ± 16 ^a^	270 ± 13 ^a^	0.34 ± 0.013 ^a,b^	0.24 ± 0.02 ^a^	8.36 ± 0.55 ^a^	17.71 ± 1.15 ^a^	0.35 ± 0.011 ^a^	0.64 ± 0.012 ^a^		
**FeSO4 0.25%**	14.00 ± 0.97 ^a,b^	11.33 ± 0.76 ^a^	279 ± 13 ^c^	239 ± 15 ^d^	0.31 ± 0.011 ^b^	0.21 ± 0.01 ^a,b^	7.51 ± 0.35 ^a,b^	14.10 ± 0.89 ^b^	0.25 ± 0.012 ^a,b^	0.41 ± 0.011 ^b^		
**FeSO4 0.5%**	14.30 ± 0.95 ^a^	10.13 ± 1.03 ^c^	285 ± 10 ^b^	246 ± 10 ^c^	0.31 ± 0.012 ^b^	0.21 ± 0.01 ^a,b^	7.70 ± 0.56 ^a,b^	14.77 ± 1.12 ^b^	0.28 ± 0.013 ^a,b^	0.42 ± 0.013 ^b^		
**LSD**	2.35		1.32		0.19		1.00		0.11			

Mean values in a column with same alphabets in superscript do not differ significantly.

**Table 2:** Influences of foliary fertigated Fe-Asp and FeSO_4_ on leaf Car, total Chl, Chl. *a*, *b*, *a*/*b*, total anthocyanin, and Leaf Relative Water Content (LRWC) of sunflower (*Halianthus annus* L.) plants when grown under limited irrigation (mean ± SE; *n* = 3).

**Treatment**	**LRWC (%)**		**Chl. *a* (mg g^−1^ FW)**		**Chl. *b* (mg g^−1^ FW)**		**Chl. *a*/*b***	
	**Non-Stressed**	**Water-Stressed**	**Non-Stressed**	**Water-Stressed**	**Non-Stress**	**Water-Stressed**	**Non-Stress**	**Water-Stressed**
**NS**	81 ± 4.00 ^c^	68 ± 3.18 ^c^	0.75 ± 0.06 ^c^	0.95 ± 0.03 ^c^	0.41 ± 0.04 ^c^	0.65 ± 0.04 ^b^	1.82 ± 0.07 ^b^	1.46 ± 0.02 ^c^
**WS**	80 ± 3.61 ^c^	67 ± 1.19 ^c^	0.82 ± 0.03 ^c^	0.98 ± 0.02 ^c^	0.45 ± 0.03 ^c^	0.66 ± 0.02 ^b^	1.82 ± 0.05 ^b^	1.48 ± 0.03 ^c^
**Fe-Asp 0.25%**	88 ± 4.50 ^a^	76 ± 3.41 ^a^	1.34 ± 0.04 ^a^	1.57 ± 0.02 ^a^	0.62 ± 0.01 ^a^	0.82 ± 0.02 ^a^	2.16 ± 0.03 ^a^	1.91 ± 0.04 ^a,b^
**Fe-Asp 0.50%**	89 ± 4.00 ^a^	75 ± 3.61 ^a^	1.31 ± 0.01 ^a^	1.61 ± 0.04 ^a^	0.66 ± 0.01 ^a^	0.83 ± 0.03 ^a^	1.98 ± 0.02 ^a^	1.95 ± 0.03 ^a^
**FeSO4 0.25%**	83 ± 2.78 ^b^	71 ± 3.81 ^b^	1.02 ± 0.08 ^b^	1.25 ± 0.03 ^b^	0.53 ± 0.03 ^b^	0.70 ± 0.02 ^b^	1.92 ± 0.03 ^b^	1.79 ± 0.02 ^a,b^
**FeSO4 0.50%**	84 ± 1.24 ^b^	72 ± 3.37 ^b^	1.07 ± 0.01 ^b^	1.21 ± 0.05 ^b^	0.56 ± 0.03 ^b^	0.71 ± 0.04 ^b^	1.91 ± 0.02 ^b^	1.70 ± 0.01 ^b^
**LSD**	1.48		0.10		0.06		0.22	
**Treatment**	**Total Chl. (mg g^−1^ FW)**		**Carotenoids (mg g^−1^ FW)**		**Anthocyanin (mg kg^−1^ FW)**			
	**Non-Stressed**	**Water-Stressed**	**Non-Stressed**	**Water-Stressed**	**Non-Stressed**	**Water-Stressed**		
**NS**	1.16 ± 0.07 ^c,d^	1.60 ± 0.02 ^d^	0.14 ± 0.07 ^d^	0.22 ± 0.02 ^d^	2.28 ± 0.03 ^d^	3.50 ± 0.13 ^d^		
**WS**	1.27 ± 0.01 ^c,d^	1.64 ± 0.04 ^c,d^	0.15 ± 0.02 ^c,d^	0.25 ± 0.04 ^c^	2.40 ± 0.12 ^c^	3.60 ± 0.02 ^c,d^		
**Fe-Asp 0.25%**	1.96 ± 0.02 ^a^	2.39 ± 0.04 ^a^	0.21 ± 0.21 ^a^	0.33 ± 0.04 ^a^	3.00 ± 0.11 ^a^	4.10 ± 0.12 ^a^		
**Fe-Asp 0.50%**	1.97 ± 0.02 ^a^	2.44 ± 0.02 ^a^	0.22 ± 0.02 ^a^	0.34 ± 0.02 ^a^	3.10 ± 0.12 ^a^	4.20 ± 0.06 ^a^		
**FeSO4 0.25%**	1.55 ± 0.01 ^b,c^	1.95 ± 0.03 ^b,c^	0.17 ± 0.01 ^b,c^	0.27 ± 0.02 ^b,c^	2.50 ± 0.07 ^b,c^	3.70 ± 0.13 ^b,c^		
**FeSO4 0.50%**	1.63 ± 0.05 ^b^	1.92 ± 0.02 ^b,c,d^	0.18 ± 0.05 ^b^	0.28 ± 0.03 ^b^	2.60 ± 0.09 ^b^	3.80 ± 0.12 ^b^		
**LSD**	0.32		0.02		0.10			

Mean values in a column with same alphabets in superscript do not differ significantly.

**Table 3:** Influences of foliary fertigated Fe-Asp and FeSO_4_ on nutrient uptake in roots and shoots of sunflower (*Halianthus annuus* L.) when grown under limited irrigation (mean ± SE; *n* = 3).

**Treatment**	**K S (mg g^−1^ DW)**		**Ca S (mg g^−1^ DW)**		**Mg S (mg g^−1^ DW)**		**P S (mg g^−1^ DW)**		**N S (mg g^−1^ DW)**		**Fe S (mg g^−1^ DW)**	
	**Non-Stressed**	**Water-Stressed**	**Non-Stressed**	**Water-Stressed**	**Non-Stressed**	**Water-Stressed**	**Non-Stressed**	**Water-Stressed**	**Non-Stressed**	**Water-Stressed**	**Non-Stressed**	**Water-Stressed**
**NS**	37 ± 0.50 ^a^	21 ± 0.76 ^c^	10.0 ± 0.26 ^d^	6.1 ± 0.29 ^c^	2.91 ± 0.26 ^c^	0.72 ± 0.33 ^c^	5.91 ± 0.27 ^c^	3.72 ± 0.33 ^c^	43.5 ± 0.50 ^b^	29.5 ± 0.76 ^b,c^	22.00 ± 0.85 ^d^	15.00 ± 0.35 ^d^
**WS**	37 ± 0.67 ^a^	22 ± 1.80 ^c^	10.3 ± 0.36 ^c,d^	6.0 ± 0.49 ^c^	3.07 ± 0.08 ^c^	0.90 ± 0.11 ^c^	6.07 ± 0.08 ^c^	3.90 ± 0.11 ^b,c^	44.0 ± 0.66 ^b^	28.5 ± 1.80 ^c^	23.00 ± 0.63 ^d^	16.00 ± 0.27 ^d^
**Fe-Asp 0.25**	38 ± 2.14 ^a^	28 ± 1.52 ^a,b^	11.0 ± 0.28 ^b^	7.9 ± 0.35 ^a^	4.10 ± 0.05 ^a^	2.18 ± 0.11 ^a^	7.10 ± 0.05 ^a^	5.18 ± 0.11 ^a^	47.0 ± 2.14 ^a^	33.0 ± 1.52 ^a^	37.17 ± 0.95 ^b^	25.43 ± 0.47 ^b,c^
**Fe-Asp 0.5**	39 ± 1.30 ^a^	29 ± 0.50 ^a^	12.0 ± 0.26 ^a^	8.3 ± 0.41 ^a^	4.47 ± 0.14 ^a^	2.10 ± 0.17 ^a^	7.47 ± 0.14 ^a^	5.10 ± 0.17 ^a^	48.0 ± 1.30 ^a^	34.5 ± 0.50 ^a^	42.50 ± 1.35 ^a^	28.00 ± 0.53 ^a^
**FeSO_4_ 0.25**	39 ± 1.69 ^a^	23 ± 2.12 ^b,c^	11.0 ± 0.28 ^b,c^	7.0 ± 0.36 ^b^	3.51 ± 0.17 ^b^	1.06 ± 0.13 ^b,c^	6.51 ± 0.17 ^b^	4.10 ± 0.13 ^b^	43.2 ± 1.69 ^b^	30.0 ± 2.12 ^b^	34.00 ± 1.01 ^c^	23.00 ± 0.49 ^c^
**FeSO_4_ 0.5**	40 ± 0.34 ^a^	24 ± 0.35 ^a,b,c^	11.3 ± 0.42 ^b,c^	7.1 ± 0.42 ^b^	3.75 ± 0.29 ^b^	1.18 ± 0.13 ^b^	6.75 ± 0.29 ^b^	4.18 ± 0.13 ^b^	44.3 ± 0.34 ^b^	31.0 ± 0.35 ^b^	36.83 ± 0.98 ^b^	25.00 ± 0.37 ^b,c^
**LSD**	5.37		0.81		0.42		0.41		1.54		2.65	
**Treatment**	**K R (mg g^−1^ DW)**		**Ca R (mg g^−1^ DW)**		**Mg R (mg g^−1^ DW)**		**P R (mg g^−1^ DW)**		**N R (mg g^−1^ DW)**		**Fe R (mg g^−1^ DW)**	
	**Non-Stressed**	**Water-Stressed**	**Non-Stressed**	**Water-Stressed**	**Non-Stressed**	**Water-Stressed**	**Non-Stressed**	**Water-Stressed**	**Non-Stressed**	**Water-Stressed**	**Non-Stressed**	**Water-Stressed**
**NS**	30.5 ± 0.50 ^b^	18.5 ± 0.76 ^b^	7.97 ± 0.16 ^b^	5.63 ± 0.29 ^b,c^	2.08 ± 0.03 ^c,e^	1.28 ± 0.04 ^b^	4.41 ± 0.26 ^d^	2.22 ± 0.13 ^c^	37.5 ± 0.5 ^b^	25.5 ± 0.76 ^c,b^	20.25 ± 0.38 ^d^	14.50 ± 0.25 ^c^
**WS**	31.3 ± 0.67 ^b^	19.0 ± 1.80 ^b^	8.33 ± 0.11 ^a,b^	5.43 ± 0.49 ^c^	2.06 ± 0.08 ^c,e^	1.30 ± 0.01 ^b^	4.57 ± 0.08 ^d^	2.40 ± 0.11 ^b,c^	36.0 ± 0.6 ^b^	24.5 ± 1.80 ^c^	21.25 ± 0.53 ^d^	15.50 ± 0.28 ^c^
**Fe-Asp 0.25**	33.7 ± 2.14 ^a^	24.0 ± 1.52 ^a^	9.00 ± 0.28 ^a,b^	6.93 ± 0.35 ^a,b^	2.50 ± 0.06 ^b^	1.69 ± 0.01 ^a^	5.60 ± 0.05 ^a,b^	3.68 ± 0.12 ^a^	40.0 ± 1.1 ^a^	30.0 ± 1.52 ^a^	31.17 ± 0.78 ^a,b^	20.00 ± 0.33 ^a,b^
**Fe-Asp 0.5**	34.3 ± 1.30 ^a^	25.0 ± 0.50 ^a^	9.50 ± 0.36 ^a^	7.27 ± 0.41 ^a^	2.75 ± 0.02 ^a^	1.71 ± 0.02 ^a^	5.97 ± 0.14 ^a^	3.60 ± 0.17 ^a^	41.0 ± 1.3 ^a^	31.0 ± 0.50 ^a^	33.00 ± 0.58 ^a^	21.50 ± 0.35 ^a^
**FeSO_4_ 0.25**	30.2 ± 1.69 ^b^	20.0 ± 2.12 ^b^	9.00 ± 0.28 ^a,b^	6.06 ± 0.16 ^a,b,c^	2.17 ± 0.02 ^c^	1.38 ± 0.01 ^b^	5.01 ± 0.17 ^c^	2.60 ± 0.13 ^b,c^	37.2 ± 1.6 ^b^	26.0 ± 2.12 ^b,c^	26.73 ± 0.45 ^c^	19.43 ± 0.58 ^b^
**FeSO_4_ 0.5**	31.3 ± 0.34 ^b^	21.7 ± 0.35 ^b^	9.50 ± 0.42 ^a^	6.18 ± 0.38 ^a,b,c^	2.13 ± 0.03 ^c^	1.36 ± 0.02 ^b^	5.25 ± 0.12 ^b,c^	2.68 ± 0.18 ^b^	37.3 ± 0.3 ^b^	27.0 ± 0.35 ^b^	30.83 ± 0.35 ^b^	19.50 ± 0.41 ^a,b^
**LSD**	2.35		1.32		0.19		0.41		1.53		2.35	

Mean values in a column with same alphabets in superscript do not differ significantly.

**Table 4:** Spearman correlation coefficients (*r*^2^) of different studied attributes of water stressed sunflower plants.

Variables	SFW	RFW	PB	NOL	LA	LRWC	C Dia	NOA	100 AW	Ach W
**SFW**	1	0.980 ***	0.990 ***	0.974 ***	0.982 ***	0.988 ***	0.985 ***	0.966 ***	0.989 ***	0.989 ***
**RFW**	0.980 ***	1	0.980 ***	0.977 ***	0.996 ***	0.946 ***	0.992 ***	0.930 ***	0.989 ***	0.992 ***
**PB**	0.990 ***	0.980 ***	1	0.978 ***	0.977 ***	0.981 ***	0.973 ***	0.966 ***	0.990 ***	0.987 ***
**SL**	0.984 ***	0.991 ***	0.987 ***	0.968 ***	0.992 ***	0.968 ***	0.989 ***	0.956 ***	0.988 ***	0.997 ***
**NOL**	0.974 ***	0.977 ***	0.978 ***	1	0.967 ***	0.947 ***	0.963 ***	0.952 ***	0.973 ***	0.973 ***
**LA**	0.982 ***	0.996 ***	0.977 ***	0.967 ***	1	0.955 ***	0.993 ***	0.931 ***	0.990 ***	0.992 ***
**LRWC**	0.988 ***	0.946 ***	0.981 ***	0.947 ***	0.955 ***	1	0.959 ***	0.974 ***	0.970 ***	0.969 ***
**C Dia**	0.985 ***	0.992 ***	0.973 ***	0.963 ***	0.993 ***	0.959 ***	1	0.942 ***	0.981 ***	0.993 ***
**NOA**	0.966 ***	0.930 ***	0.966 ***	0.952 ***	0.931 ***	0.974 ***	0.942 ***	1	0.946 ***	0.962 ***
**100 AW**	0.989 ***	0.989 ***	0.990 ***	0.973 ***	0.990 ***	0.970 ***	0.981 ***	0.946 ***	1	0.992 ***
**Ach W**	0.989 ***	0.992 ***	0.987 ***	0.973 ***	0.992 ***	0.969 ***	0.993 ***	0.962 ***	0.992 ***	1
**Chl. a**	−0.046 ns	−0.204 ns	−0.057 ns	−0.088 ns	−0.200 ns	0.055 ns	−0.143 ns	0.139	−0.130	−0.109 ns
**Chl. b**	−0.491**	−0.619 ***	−0.494**	−0.512 ***	−0.616 ***	−0.393*	−0.567 ***	−0.302**	−0.567 ***	−0.538 ***
**T Chl.**	−0.194 ns	−0.346**	−0.202 ns	−0.230 ns	−0.343**	−0.091 ns	−0.286**	−0.003 ns	−0.278 ns	−0.254 ns
**Chl. a/b**	0.811 ***	0.713 ***	0.790 ***	0.751 ***	0.720 ***	0.846 ***	0.748 ***	0.847 ***	0.774 ***	0.765 ***
**Car**	−0.552 ***	−0.667 ***	−0.550 ***	−0.564 ***	−0.674 ***	−0.471**	−0.624 ***	−0.379**	−0.612 ***	−0.595 ***
**LRMP**	−0.989 ***	−0.967 ***	−0.984 ***	−0.964 ***	−0.966 ***	−0.983 ***	−0.979 ***	−0.971 ***	−0.970 ***	−0.980 ***
**MDA**	−0.947 ***	−0.949 ***	−0.924 ***	−0.890 ***	−0.953 ***	−0.915 ***	−0.958 ***	−0.853 ***	−0.950 ***	−0.944 ***
**H2O2**	−0.948 ***	−0.927 ***	−0.923 ***	−0.904 ***	−0.920 ***	−0.921 ***	−0.946 ***	−0.899 ***	−0.929 ***	−0.935 ***
**TPC**	0.980 ***	0.956 ***	0.960 ***	0.957 ***	0.950 ***	0.955 ***	0.965 ***	0.941 ***	0.965 ***	0.964 ***
**AsA**	0.980 ***	0.956 ***	0.960 ***	0.957 ***	0.950 ***	0.955 ***	0.965 ***	0.941 ***	0.965 ***	0.964 ***
**Antho**	−0.688 ***	−0.786 ***	−0.678 ***	−0.689 ***	−0.790 ***	−0.607 ***	−0.755 ***	−0.520 ***	−0.738 ***	−0.723 ***
**F antho**	−0.760 ***	−0.838 ***	−0.761 ***	−0.741 ***	−0.854 ***	−0.714 ***	−0.818 ***	−0.633 ***	−0.804 ***	−0.800 ***
**Flav**	−0.742 ***	−0.824 ***	−0.740 ***	−0.728 ***	−0.840 ***	−0.692 ***	−0.801 ***	−0.614 ***	−0.786 ***	−0.782 ***
**CAT**	−0.690 ***	−0.777 ***	−0.707 ***	−0.686 ***	−0.784 ***	−0.634 ***	−0.731 ***	−0.541 ***	−0.754 ***	−0.728 ***
**POD**	−0.681 ***	−0.778 ***	−0.702 ***	−0.680 ***	−0.784 ***	−0.625 ***	−0.735 ***	−0.547 ***	−0.745 ***	−0.730 ***
**APX**	−0.571 ***	−0.689 ***	−0.570 ***	−0.579 ***	−0.698 ***	−0.494**	−0.650 ***	−0.418**	−0.637 ***	−0.628 ***
**SOD**	−0.769 ***	−0.837 ***	−0.760 ***	−0.739 ***	−0.851 ***	−0.716 ***	−0.816 ***	−0.622 ***	−0.812 ***	−0.798 ***
**Gluta**	−0.699 ***	−0.795 ***	−0.682 ***	−0.695 ***	−0.802 ***	−0.620 ***	−0.772 ***	−0.540 ***	−0.744 ***	−0.736 ***
**Asp**	0.134 ns	0.167 ns	0.185 ns	0.270 ns	0.124 ns	0.090 ns	0.100 ns	0.116 ns	0.144 ns	0.098 ns
**Lys**	−0.496**	−0.593 ***	−0.480**	−0.445**	−0.616 ***	−0.436**	−0.575 ***	−0.320**	−0.549 ***	−0.539 ***
**Meth**	−0.882 ***	−0.945 ***	−0.884 ***	−0.922 ***	−0.944 ***	−0.829 ***	−0.923 ***	−0.813 ***	−0.906 ***	−0.910 ***
**Pro**	−0.128 ns	−0.119 ns	−0.173 ns	−0.104 ns	−0.113 ns	−0.153 ns	−0.097 ns	−0.250 ns	−0.148 ns	−0.162 ns
**GB**	−0.748 ***	−0.832 ***	−0.734 ***	−0.729 ***	−0.844 ***	−0.684 ***	−0.815 ***	−0.599 ***	−0.790 ***	−0.784 ***
**GSSG**	−0.804 ***	−0.871 ***	−0.803 ***	−0.781 ***	−0.882 ***	−0.752 ***	−0.846 ***	−0.668 ***	−0.849 ***	−0.834 ***
**GSG**	−0.559 ***	−0.657 ***	−0.558 ***	−0.530 ***	−0.671 ***	−0.496**	−0.624 ***	−0.374**	−0.619 ***	−0.595 ***
**FAA**	−0.993 ***	−0.986 ***	−0.979 ***	−0.964 ***	−0.991 ***	−0.976 ***	−0.996 ***	−0.951 ***	−0.986 ***	−0.992 ***
**TSP**	−0.439**	−0.571 ***	−0.461**	−0.455**	−0.573 ***	−0.359**	−0.513 ***	−0.263 ns	−0.523 ***	−0.494 ***
**TSS**	−0.725 ***	−0.821 ***	−0.730 ***	−0.727 ***	−0.828 ***	−0.659 ***	−0.784 ***	−0.581 ***	−0.783 ***	−0.767 ***
**R. Sug**	−0.662 ***	−0.771 ***	−0.682 ***	−0.676 ***	−0.775 ***	−0.593 ***	−0.720 ***	−0.523 ***	−0.734 ***	−0.713 ***
**NRS**	−0.774 ***	−0.852 ***	−0.762 ***	−0.763 ***	−0.864 ***	−0.713 ***	−0.833 ***	−0.627 ***	−0.815 ***	−0.805 ***
**KS**	0.980 ***	0.983 ***	0.959 ***	0.957 ***	0.984 ***	0.948 ***	0.993 ***	0.926 ***	0.973 ***	0.980 ***
**Ca S**	0.991 ***	0.973 ***	0.972 ***	0.952 ***	0.976 ***	0.977 ***	0.989 ***	0.953 ***	0.972 ***	0.983 ***
**Mg S**	0.988 ***	0.962 ***	0.980 ***	0.959 ***	0.968 ***	0.990 ***	0.977 ***	0.982 ***	0.969 ***	0.982 ***
**NS**	0.989 ***	0.992 ***	0.987 ***	0.974 ***	0.994 ***	0.968 ***	0.987 ***	0.948 ***	0.993 ***	0.994 ***
**PS**	0.988 ***	0.962 ***	0.981 ***	0.959 ***	0.968 ***	0.991 ***	0.977 ***	0.982 ***	0.969 ***	0.982 ***
**K R**	0.993 ***	0.986 ***	0.993 ***	0.975 ***	0.984 ***	0.977 ***	0.984 ***	0.969 ***	0.993 ***	0.995 ***
**Ca R**	0.955 ***	0.912 ***	0.954 ***	0.916 ***	0.925 ***	0.980 ***	0.928 ***	0.975 ***	0.935 ***	0.948 ***
**Mg R**	0.975 ***	0.951 ***	0.985 ***	0.964 ***	0.952 ***	0.982 ***	0.953 ***	0.977 ***	0.963 ***	0.971 ***
**N R**	0.997 ***	0.982 ***	0.992 ***	0.985 ***	0.983 ***	0.983 ***	0.980 ***	0.964 ***	0.992 ***	0.989 ***
**P R**	0.988 ***	0.962 ***	0.981 ***	0.959 ***	0.968 ***	0.991 ***	0.977 ***	0.982 ***	0.969 ***	0.982 ***
**Fe S**	0.918 ***	0.866 ***	0.894 ***	0.846 ***	0.890 ***	0.946 ***	0.903 ***	0.921 ***	0.884 ***	0.908 ***
**Fe R**	0.817 ***	0.721 ***	0.807	0.739 ***	0.741 ***	0.882 ***	0.760 ***	0.871 ***	0.774 ***	0.792 ***

***, ** and * = significant at 0.001, 0.01 and 0.05 levels respectively.

## Abbreviations

RFW	root fresh weight
SFW	shoot fresh weight
PB	plant biomass
SL	shoot length
NOL	number of leaves
LA	leaf area
LRWC	leaf relative water content
C Dia	capitulum diameter
NOA	number of achenes per capitulum
100 AW	100 achene weight
Ach W	achene weight per capitulum
Chl. b	chlorophyll b
Chl. *a*	chlorophyll a
T Chl	total chlorophyll
Chl. a/b	chlorophyll a/b ratio
Car	carotenoids
LRMP	leaf relative membrane permeability
MDA	malondialdehyde
H_2_O_2_	hydrogen peroxide
TPC	total phenolic content
AsA	ascorbic acid
Antho	anthocyanin
Flav	flavonoids
POD	peroxidase
SOD	superoxide dismutase
CAT	catalase
APX	ascorbate peroxidase
Gluta	glutathione
Meth	methionine
Lys	lysine
Asp	aspartate
GB	glycine betaine
Pro	proline
GSG	reduced glutathione
GSSG	oxidized glutathione
FAA	free amino acids
TSP	total soluble proteins
TSS	total soluble sugars
R Sug	redusing sugars
NRS	non-reducing sugars
KS	shoot K
Ca S	shoot Ca
Mg S	shoot Mg
NS	shoot N
PS	shoot P
N R	root N
Mg R	root Mg
K R	root K
Ca R	root Ca
P R	root P
Fe S	shoot Fe
Fe R	root Fe
